# Improving Log-Likelihood Ratio Estimation with Bi-Gaussian Approximation under Multiuser Interference Scenarios

**DOI:** 10.3390/e23060784

**Published:** 2021-06-20

**Authors:** Yu Fu, Hongwen Yang

**Affiliations:** School of Information and Communication Engineering, Beijing University of Posts and Telecommunications, Beijing 100876, China; yanghong@bupt.edu.cn

**Keywords:** bi-Gaussian approximation, log-likelihood ratio, multiuser interference, LDPC codes, word error rate, decoding complexity

## Abstract

Accurate estimation of channel log-likelihood ratio (LLR) is crucial to the decoding of modern channel codes like turbo, low-density parity-check (LDPC), and polar codes. Under an additive white Gaussian noise (AWGN) channel, the calculation of LLR is relatively straightforward since the closed-form expression for the channel likelihood function can be perfectly known to the receiver. However, it would be much more complicated for heterogeneous networks where the global noise (i.e., noise plus interference) may be dominated by non-Gaussian interference with an unknown distribution. Although the LLR can still be calculated by approximating the distribution of global noise as Gaussian, it will cause performance loss due to the non-Gaussian nature of global noise. To address this problem, we propose to use bi-Gaussian (BG) distribution to approximate the unknown distribution of global noise, for which the two parameters of BG distribution can easily be estimated from the second and fourth moments of the overall received signals without any knowledge of interfering channel state information (CSI) or signaling format information. Simulation results indicate that the proposed BG approximation can effectively improve the word error rate (WER) performance. The gain of BG approximation over Gaussian approximation depends heavily on the interference structure. For the scenario of a single BSPK interferer with a 5 dB interference-to-noise ratio (INR), we observed a gain of about 0.6 dB. The improved LLR estimation can also accelerate the convergence of iterative decoding, thus involving a lower overall decoding complexity. In general, the overall decoding complexity can be reduced by 25 to 50%.

## 1. Introduction

The discovery of turbo codes [1,2], low-density parity-check (LDPC) codes [3,4], and polar codes [5] represent major milestones in channel coding. These codes are said to be “near capacity achieving” codes due to their excellent performance; they can nearly reach the Shannon limit [6] and have been incorporated into various communication standards [7,8,9,10,11]. The decoding of these codes generally adopts the so called “soft-input decoding” where the input of the decoder is the bit-level log-likelihood ratio (LLR), which is a probabilistic metric indicating how likely it is that the underlying code bit is “1” or “0”. LLR can be calculated with the channel likelihood function, p(y|x), i.e., the transfer probability density function (PDF) between the channel input *x* and the output *y*.

There have been extensive studies devoted to LLR evaluation. For the additive white Gaussian noise (AWGN) channel, the channel likelihood function is Gaussian and the studies mainly focus on the low-complexity calculation of LLR. For example, ref. [12] described a simplified LLR approximation for high-order modulations and [13] proposed a low-complexity LLR computation for nonuniform PAM constellations. To reduce the LLR calculation complexity of 64APK, ref. [14] proposed an algorithm by taking full advantage of the symmetric characteristics of symbol mapping. For massive-order non-uniform constellations, low-complexity demapping algorithms were proposed in [15,16] for one- and two-dimension constellations, respectively, and [17] proposed a universal low-complexity demapper for non-uniform constellations. For index modulation, a low-complexity LLR calculation algorithm was proposed in [18].

In addition, many communication channels have exhibited non-Gaussian channel likelihood functions, due to the non-Gaussian additive or multiplicative noise. For example, in power line communication (PLC), the impulsive noise can be characterized by the Bernoulli–Gaussian model [19,20] or the Middleton Class-A model [21,22]. Research in [23,24] proposed some algorithms for adaptive demodulation in impulse noise channels. The proposed methods compute appropriate LLRs based on four previously established parameter estimation techniques of symmetric α-stable noise and the classification or parameter estimation of Middleton’s Class A noise. The non-Gaussian model has also appeared in multiuser/multi-antenna communication systems where the interference is generally non-Gaussian [25,26].

The exact evaluation of LLR requires knowledge about the underlying channel model or noise model [27]. On a fading channel with no channel state information (CSI) at the receiver, true log-likelihood ratios are complicated functions of the channel output. To address this problem, ref. [28] proposed a linear LLR approximation whose performance is extremely close to that of the true LLR calculation on an uncorrelated Rayleigh fading channel. Ref. [29] presented a greedy algorithm for data detection in uplink grant-free non-orthogonal multiple access (NOMA), which requires no knowledge of noise variance by computing the LLR approximately in its operation. For the non-Gaussian noise, the assumption of perfect knowledge of noise statistics at the receiver end might be impractical and hence the estimation of noise parameters is necessary. For the Middleton Class-A impulsive noise in PLC, a deep learning approach is proposed in [30] to estimate the distribution parameters. Alternatively, deep-learning-based decoding can be used for channels with non-Gaussian noise. Ref. [31] proposed a neural architecture for turbo decoding, which replaces the Bahl–Cocke–Jelinek–Raviv (BCJR) algorithm with a bidirectional gated recurrent unit (Bi-GRU). Ref. [32] improved the neural BCJR and showed an end-to-end trained decoder named DEEP TURBO. Both [31,32] have shown good adaptability under some non-Gaussian settings, and the LLR calculation is implicitly implemented inside the neural network. Several attempts have also been made to solve the unknown multiuser interference. Ref. [26] proposed improving the turbo-decoding scheme with an iterative PDF estimator. A kernel-based method is used to estimate the PDF of global noise from the received signals. There are two iterative processes in this scheme. One is the iterative process within the turbo decoder, and the other is the iterative feedback to refine the global noise samples for the PDF estimator. Similarly, ref. [33] proposed one-bit successive-cancellation soft-output (OSS) detectors for an uplink multiuser system, which can exploit the a priori information conveyed by channel decoders to improve the LLRs. It also adopts the iterative feedback of the previously decoded messages. Refs. [25,34] coupled a multiuser detector and single-user turbo decoders. After each turbo decoding iteration, the extrinsic information of interfering users is passed to the multiuser detector, and each multiuser iteration passes the updated a posteriori probabilities to single-user turbo decoders. A joint iterative channel estimation and multiuser detection technique is proposed in [35] for overloaded multiple-input multiple-output (MIMO) orthogonal frequency division multiplexing (OFDM) systems. The channel estimator, the detector, and the decoder work like an iterative cycle, through which more reliable LLRs are updated.

In this paper, we focus on the scenario where a point-to-point communication is polluted by both the Gaussian noise and the non-Gaussian interference from nearby stations. This scenario may be typical for today’s heterogeneous networks consisting of an overlay of several dense, irregularly, and often completely randomly deployed networks with a limited coverage area [36]. For such complicated networks, the overhead of acquiring the CSI of the interfering channel and the modulation and coding scheme (MCS) of interfering signals may be impractically high, implying that the joint detection or successive interference cancellation may not be possible at least for some situations, especially when the interfering station belongs to a different system or operator. In these cases, the global noise is typically treated as Gaussian noise [37,38,39,40]. However, since interfering signals are drawn from a finite constellation as the desired signal, the real distribution is definitely non-Gaussian. This implies that the existing method (i.e., approximating the global noise as Gaussian for LLR estimation) may incur some performance loss.

To address this problem, we propose to use bi-Gaussian (BG) approximation instead of Gaussian approximation for the LLR estimation. Specifically, the contributions of this paper include the following:

1. For the scenario where the global noise consists of Gaussian noise and multiuser interference and the CSI and MSC information of interfering signals are unknown, a bi-Gaussian distribution is proposed to approximate the global noise;
2. A simple algorithm is proposed to estimate the two parameters of the BG distribution;
3. The BG distribution together with the estimated parameters are then used to calculate the LLR;
4. We have conducted simulations to verify the advantages of the proposed BG approximation (BGA) over the existing Gaussian approximation (GA) and the results show that BGA outperforms GA in both WER performance and decoding complexity.

The rest of this paper is organized as follows. Section 2 introduces the system model and the LLR computation. Section 3 proposes the bi-Gaussian approximation and the parameter estimation, together with the LLR estimation. Section 4 presents the simulation results and comparisons of WER and complexity. Section 5 concludes the paper.

## 2. System Model

**Figure 1:**
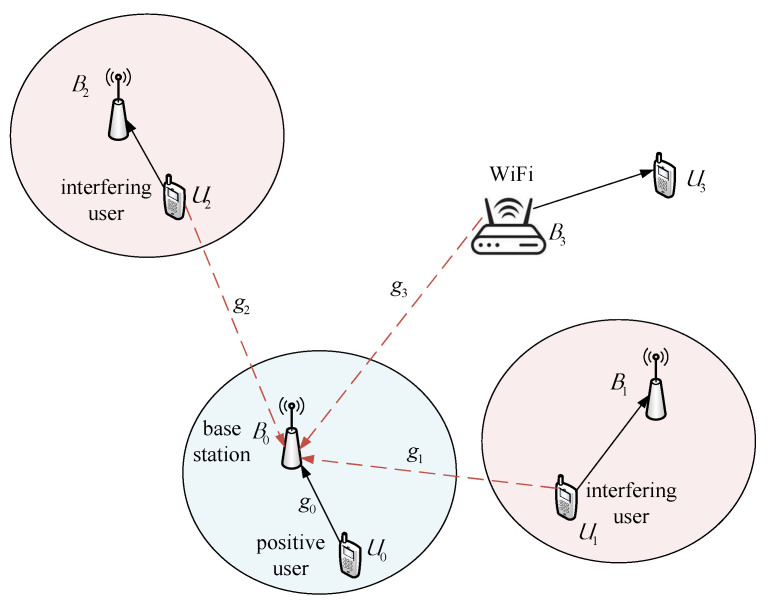
Multiuser interfering model.

Consider an example communication scenario shown in Figure 1. User $U_{0}$ is transmitting a signal to its base station $B_{0}$. At the same time, user $U_{1}$ and user $U_{2}$ are transmitting their signals to their respective base stations $B_{1},B_{2}$. In addition, an access point $B_{3}$ is transmitting a signal to terminal $U_{3}$ (i.e., $U_{0},U_{1},U_{2}$ are hidden nodes to $B_{3}$ and $U_{3}$). It is possible that all radio links shown in Figure 1 may share the same frequency spectrum. This implies that the signal transmitted by $U_{0}$ will receive interference by way of signals from $U_{1},U_{2}$ and $B_{3}$. At base station $B_{0}$, the way of treating interference will directly affect the receiving performance.

It is obvious that the optimal processing at $B_{0}$ is to jointly detect all signals from $U_{0},U_{1},U_{2}$, and $B_{3}$ [41,42]. To this end, the signals arriving at $B_{0}$ must be synchronized in time and the CSI of the channels from $U_{0},U_{1},U_{2},B_{3}$ to $B_{0}$ must be known to $B_{0}$. Moreover, $B_{0}$ must be informed of the signaling format including MCS. On the contrary, one can approximate all the interference as Gaussian noise. Such Gaussian approximation can greatly reduce the complexity of detection but it will degrade the detection performance as well.

### 2.1. Global Noise

With *K* interferers, the signal observed at target base station $B_{0}$ can be written as

(1) $$y=\sqrt{g_{0}}x_{0}+\sum_{k=1}^{K}\sqrt{g_{k}}x_{k}+w,$$

where $x_{0}$ is the desired signal of $U_{0}$, $g_{0}$ is the channel gain from target user $U_{0}$ to $B_{0}$, $x_{1},\cdots ,x_{K}$ are the interfering signals, and $g_{1},g_{2},\cdots ,g_{K}$ are the channel gains from *k*-th interferer to $B_{0}$ ( For the reason of convenience, we have assumed the block fading channel where $g_{1},\cdots ,g_{K}$ does not change within the duration of one codeword. The point of this paper is that the global noise $\sum_{k=1}^{K}\sqrt{g_{k}}x_{k}+w$ can be better approximated by bi-Gaussian than by Gaussian. This idea is obviously applicable to the situation where $g_{1},\cdots ,g_{K}$ may change within codeword duration (e.g., the subcarriers of the OFDM system may undergo frequency selective fading)), and $w∼N(0,\sigma^{2})$ is the additive white Gaussian noise. $N(\mu ,\sigma^{2})$ denotes a Gaussian distribution with mean μ and variance $\sigma^{2}$.

We assume that $g_{0}$ is perfectly known to $B_{0}$. Under this assumption, the coefficient $g_{0}$ can be scaled off with an ideal automatic gain control (AGC), and hence we assume $g_{0}=1$ hereafter.

From the perspective of $B_{0}$, only $g_{0}=1$ on the right-hand-side (RHS) of (1) is known and all remaining variables are random. We assume that random variables $x_{0},x_{1},\cdots ,x_{K},g_{1},\cdots ,g_{K}$ and *w* are mutually independent.

For simplicity, we assume that $x_{0}$ is binary phase shift keying (BPSK) modulated and $x_{0}\in \left\{\pm 1\right\}$ with equal probability. We also assume that $E[x_{k}^{2}]=1$ for k=1,2,⋯,K. Note that although different interferers may have different signal powers, this factor can be included in the channel gains $\left\{g_{k}\right\}$.

Based on (1), the signal-to-interference-plus-noise ratio (SINR) and interference-to-noise ratio (INR) [43,44] can be calculated as

(2) $$\mathrm{SINR}=\frac{1}{\sum_{k=1}^{K}g_{k}+\sigma^{2}},$$

and

(3) $$\mathrm{INR}=\frac{\sum_{k=1}^{K}g_{k}}{\sigma^{2}},$$

respectively.

In this paper, we refer to the aggregate of interference plus noise as *global noise*, which is

(4) $$z=\sum_{k=1}^{K}\sqrt{g_{k}}x_{k}+w.$$

The distribution of global noise *z* depends on the distribution of $x_{1},\cdots ,x_{K},g_{1},\cdots ,g_{K}$ and *w*. In general, it would be difficult to find a closed-form expression for the PDF of *z*, or the PDF is intractably complicated. For example, even if $g_{1},\cdots ,g_{K}$ are known to $B_{0}$, and if $x_{1},\cdots ,x_{K}$ are all BPSK symbols, then the PDF is the mixed Gaussian distribution given by

(5) $$p_{z}\left(z\right)=\frac{1}{2^{K}\sqrt{2\pi \sigma^{2}}}\sum_{x_{1},x_{2},\cdots ,x_{K}\in {\left\{\pm 1\right\}}^{K}}e^{-\frac{{\left(z-\sum_{k=1}^{K}\sqrt{g_{k}}x_{k}\right)}^{2}}{2\sigma^{2}}},$$

where ${\left\{\pm 1\right\}}^{K}$ refers to the Cartesian power of set {−1,+1}. In practice, this distribution (5) is generally considered too complex for the LLR calculation since the number of terms in the summation increases exponentially with the number of interferers. What is worse is that, when $g_{1},g_{2},\cdots ,g_{K}$ are random variables, the PDF of global noise *z* may have no closed-form expression even with very simple fading models for $\left\{g_{k}\right\}$.

### 2.2. Soft Demapping

**Figure 2:**
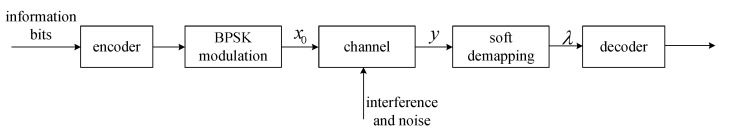
Soft demapping: the input of the decoder is bit-level LLR.

We assume that the signal of $U_{0}$ is encoded by a binary channel code like turbo, LDPC, polar, or convolutional code. To attain maximum coding gain, the received signal *y* is first converted to a soft metric λ, and then fed to the soft-input decoder, as illustrated in Figure 2.

The soft metric λ is conventionally referred to as LLR, which is actually defined as the logarithm of the a posteriori probability ratio:

(6) $$\lambda =\ln\frac{\mathrm{Pr}\{x_{0}=+1|y\}}{\mathrm{Pr}\{x_{0}=-1|y\}}=\ln\frac{p_{z}\left(y-1\right)}{p_{z}\left(y+1\right)}.$$

In case the global noise is Gaussian, i.e., $z∼N(0,\sigma_{G}^{2})$, the soft-demapping reduces to a scaling operation:

(7) $$\lambda_{G}=\ln\frac{p_{G}\left(y-1\right)}{p_{G}\left(y+1\right)}=\frac{2y}{{\sigma_{G}}^{2}},$$

where $p_{G}\left(z\right)$ denotes the PDF of $N(0,{\sigma_{G}}^{2})$.

The global noise defined in (4) is generally non-Gaussian. If $g_{1},\cdots ,g_{K}$ are known to $B_{0}$ and $x_{1},\cdots ,x_{K}$ are all BPSK symbols, the non-Gaussian PDF is given by (5). Substituting (5) into the RHS of (6), the soft metric is calculated as

(8) $$\lambda =\ln\frac{\sum_{x_{1},x_{2},\cdots ,x_{K}\in {\left\{\pm 1\right\}}^{K}}e^{-\frac{{\left(y-1-\sum_{k=1}^{K}\sqrt{g_{k}}x_{k}\right)}^{2}}{2\sigma^{2}}}}{\sum_{x_{1},x_{2},\cdots ,x_{K}\in {\left\{\pm 1\right\}}^{K}}e^{-\frac{{\left(y+1-\sum_{k=1}^{K}\sqrt{g_{k}}x_{k}\right)}^{2}}{2\sigma^{2}}}}.$$

It is obvious that the computational complexity of (8) is much higher than (7), and yet the computational complexity of (8) has not taken into account the complexity spent on the channel estimation of $g_{1},g_{2},\cdots ,g_{K}$. Moreover, in some scenarios, some of the interferers may belong to different operators, and some of the interferers may belong to an unknown system with different air–interface protocols. In such cases, it would be very hard for $B_{0}$ to accurately estimate all $g_{1},g_{2},\cdots ,g_{K}$, and even the number of interferers *K* may be unknown to $B_{0}$.

In view of these problems, we propose to approximate the PDF of global noise as a bi-Gaussian distribution, which will be elaborated in the next section.

## 3. LLR Estimation with Bi-Gaussian Approximation

### 3.1. Bi-Gaussian Distribution

In this paper, we use the term *bi-Gaussian distribution* [45,46] to refer to the symmetrical mixed-Gaussian distribution for which the PDF is given by

(9) $$\begin{array}{cc} p_{\mathrm{BG}}\left(z\right) & =\frac{1}{2\sqrt{2\pi \sigma^{2}}}e^{-\frac{{\left(z-\mu \right)}^{2}}{2\sigma^{2}}}+\frac{1}{2\sqrt{2\pi \sigma^{2}}}e^{-\frac{{\left(z+\mu \right)}^{2}}{2\sigma^{2}}}\\ \; & =\frac{1}{\sqrt{2\pi \sigma^{2}}}e^{-\frac{z^{2}+\mu^{2}}{2\sigma^{2}}}\cosh\left(\frac{\mu z}{\sigma^{2}}\right). \end{array}$$

It can be noted that (9) is a special case of (5) with $K=1,\sqrt{g_{1}}=\mu$. In other words, the bi-Gaussian distribution is the distribution of the global noise with single BPSK interference.

The bi-Gaussian distribution is used in [45] where a shifted bi-Gaussian mixture model is introduced to match the image intensity histogram. In addition, in [46], the bi-Gaussian function is proposed to replace the low-level Gaussian kernel in derivative filters for image segmentation and enhancement. Bi-Gaussian distribution also appears in [47], where an analytical expression is developed for the differential entropy of this distribution. In this paper, we will use (9) to approximate the distribution of global noise with multiuser interferences.

Due to the symmetry of a bi-Gaussian PDF, all the odd moments are zero. The second moment (variance) and fourth moment are listed as follows

(10) $$\begin{array}{cc} \mu_{2}^{\mathrm{BG}} & =\mu^{2}+\sigma^{2},\\ \mu_{4}^{\mathrm{BG}} & =\mu^{4}+3\sigma^{4}+6\mu^{2}\sigma^{2}. \end{array}$$

### 3.2. Bi-Gaussian Approximation

Since the exact distribution of global noise *z* is either too complicated for LLR calculation if $g_{1},g_{2},\cdots ,g_{K}$ are known, or is intractable if $g_{1},g_{2},\cdots ,g_{K}$ are unknown, we have to resort to the approximate distribution to perform the calculation in (6). The popular Gaussian approximation regards the global noise as the Gaussian noise with distribution $N(0,\sigma_{z}^{2})$, where $\sigma_{z}^{2}=E\left[z^{2}\right]$. E[·] denotes the expectation operation. Gaussian approximation has the advantage of computational simplicity, but the cost is performance degradation. This is because Gaussian noise has the worst differential entropy for a given noise power. In cases where the global noise consists of Gaussian noise and multiple BPSK interference, the entropy power of global noise can be much less than the real power $\sigma_{z}^{2}$ [48].

By considering the trade-off between complexity and performance, we use a bi-Gaussian distribution to approximate the distribution of global noise. The approximation is based on the equivalence in terms of variance and kurtosis, i.e.,

(11) $$\begin{array}{cc} \sigma_{z}^{2} & =\mu_{2}^{\mathrm{BG}},\\ \kappa_{z} & =\frac{\mu_{4}^{\mathrm{BG}}}{{\left(\mu_{2}^{\mathrm{BG}}\right)}^{2}}, \end{array}$$

where $\kappa_{z}=E\left[z^{4}\right]/\sigma_{z}^{4}$.

Equation (11) implies that, with bi-Gaussian approximation, the receiver is not required to estimate the CSI of each individual interferer. The overall noise power and noise kurtosis are sufficient to solve the parameters of bi-Gaussian distribution. The solution is given by

(12) $$\begin{array}{cc} \mu & =\sigma_{z}\sqrt[{4}]{\frac{3-\kappa_{z}}{2}},\\ \sigma^{2} & =\sigma_{z}^{2}-\sigma_{z}^{2}\sqrt{\frac{3-\kappa_{z}}{2}}. \end{array}$$

The power $\sigma_{z}^{2}$ and the kurtosis $\kappa_{z}$ of global noise *z* can be estimated from the second and fourth moments of *y*. Since $y=x_{0}+z$, the moments $E[z^{2}]$ and $E[z^{4}]$ can be expressed by $E[y^{2}]$ and $E[y^{4}]$ as

(13) $$\begin{array}{cc} E[z^{2}] & =E[y^{2}]-1,\\ E[z^{4}] & =E\left[y^{4}\right]-6E\left[y^{2}\right]+5. \end{array}$$

Further, the second and fourth moments of *y* can be estimated through sample averaging of the received signal vector. Let *N* be the length of a codeword, and $y=(y_{1},y_{2},\cdots ,y_{N})$ be the received signals corresponding to one codeword, then the second and fourth moments of *y* can be estimated by

(14) $$\begin{array}{cc} E[y^{2}] & \approx \frac{1}{N}\sum_{i=1}^{N}{y_{i}}^{2},\\ E[y^{4}] & \approx \frac{1}{N}\sum_{i=1}^{N}{y_{i}}^{4}. \end{array}$$

Finally, the parameters $\mu ,\sigma^{2}$ of bi-Gaussian approximation can be obtained directly from the received signals before detection. The specific steps are as follows. (1) $E[y^{2}]$ and $E[y^{4}]$ are estimated from the received signals through (14). (2) $E[z^{2}]$ and $E[z^{4}]$ can be calculated using (13). Then $\sigma_{z}^{2}$ and $\kappa_{z}$ are obtained. (3) Parameters $\mu ,\sigma^{2}$ are finally estimated using (12).

**Figure 3:**
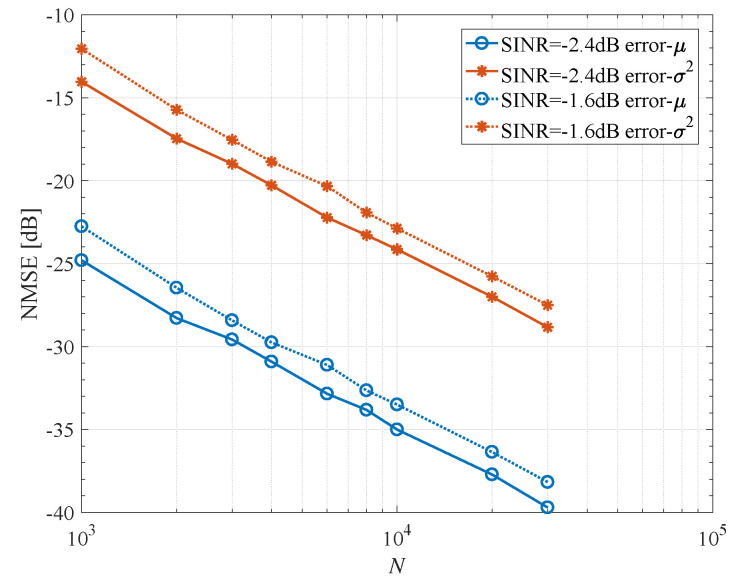
NMSE of parameter estimation. K=1, INR = 5 dB.

The normalized mean square error (NMSE) of estimated parameters $\mu ,\sigma^{2}$ is given in Figure 3 for K=1 and INR = 5 dB. We can see that the estimation method proposed above is satisfactory. A longer codeword (or estimation with several successive codewords) will have better estimation.

**Figure 4:**
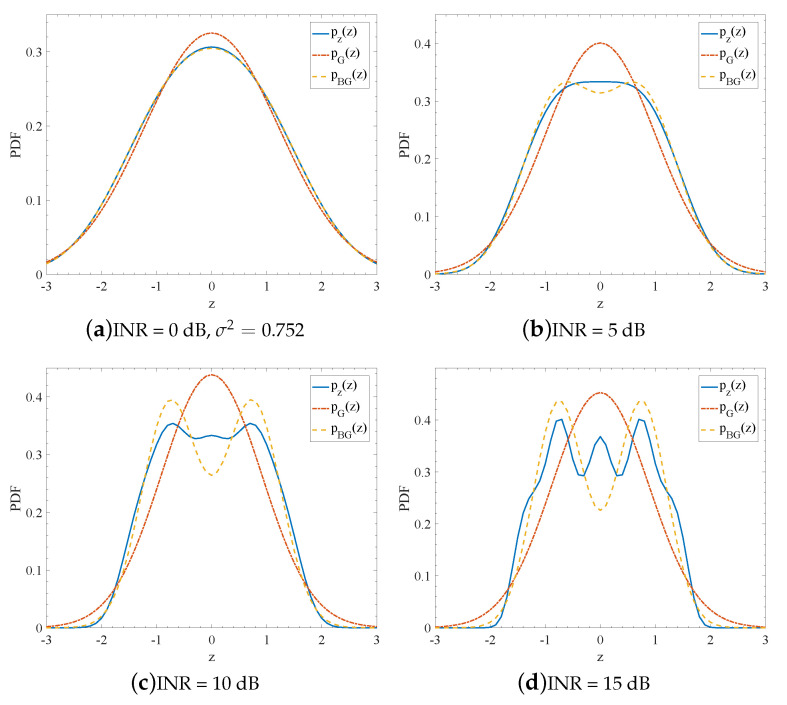
PDF of actual distribution $p_{z}\left(z\right)$, Gaussian approximation $p_{G}\left(z\right)$, and bi-Gaussian approximation $p_{\mathrm{BG}}\left(z\right)$ under conditions K=5, $g_{1}=0.549$, $g_{2}=0.124$, $g_{3}=0.057$, $g_{4}=0.015$, and $g_{5}=0.007$.

Figure 4 depicts an example for the PDF of the real distribution $p_{z}\left(z\right)$, the Gaussian approximation $p_{G}\left(z\right)$, and the bi-Gaussian approximation $p_{\mathrm{BG}}\left(z\right)$ under conditions K=5, $g_{1}=0.549$, $g_{2}=0.124$, $g_{3}=0.057$, $g_{4}=0.015$, and $g_{5}=0.007$. The Gaussian approximation has the same noise power as the real global noise and the bi-Gaussian approximation has the same noise power and kurtosis as the real global noise. We can see that bi-Gaussian approximation looks more similar to the true distribution than the Gaussian approximation, especially when the INR is large.

Apparently, considering more terms in the mixed Gaussian distribution (5) can better approximate the true PDF of global noise. For example, with four terms, the four-Gaussian (FG) distribution is given by

(15) $$p_{\mathrm{FG}}\left(z\right)=\frac{1}{4\sqrt{2\pi \sigma^{2}}}\left\{e^{-\frac{{\left(z-\mu_{1}\right)}^{2}}{2\sigma^{2}}}+e^{-\frac{{\left(z-\mu_{2}\right)}^{2}}{2\sigma^{2}}}+e^{-\frac{{\left(z+\mu_{1}\right)}^{2}}{2\sigma^{2}}}+e^{-\frac{{\left(z+\mu_{2}\right)}^{2}}{2\sigma^{2}}}\right\}.$$

which has 3 parameters $\mu_{1},\mu_{2},\sigma^{2}$. We will show later that the gain from including more terms is quite limited, while the cost can be much larger—as the number of terms in the mixed Gaussian distribution increases, the number of parameters to be estimated also increases and so is the calculation complexity of LLR.

**Figure 5:**
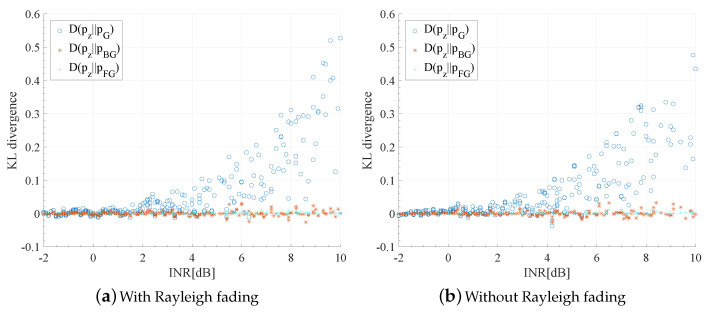
KL divergence between actual distribution $p_{z}\left(z\right)$ and Gaussian approximation $p_{G}\left(z\right)$, bi-Gaussian approximation $p_{\mathrm{BG}}\left(z\right)$, and four-Gaussian approximation $p_{\mathrm{FG}}\left(z\right).$

In Figure 5, we show the Kullback–Leibler (KL) divergence of Gaussian approximation, bi-Gaussian approximation, and four-Gaussian approximation with respect to the true distribution $p_{z}\left(z\right)$. KL divergence, also known as relative entropy [49], is an asymmetric measure of the difference between two probability distributions. The KL divergence of two distributions p,q is defined as

(16) $$D\left(p||q\right)≜\int p(x)\log\frac{p(x)}{q(x)}dx.$$

The results were obtained through Monte Carlo simulation under condition K=5 and randomly generated $g_{1},g_{2},\cdots ,g_{K}$. In the simulation, the locations of interferers are uniformly drawn within an area surrounding the receiver $B_{0}$. The interfering path gain $g_{k},k=1,2,\cdots ,K$ is determined by $g_{k}=\beta h_{k}d_{k}^{-\alpha }$, where α=4 is the path loss exponent, $d_{k}$ is the distance from *k*-th interferer to the receiver $B_{0}$, $h_{k}$ represents the small-scale fading, and β is a normalizing coefficient for the sake of satisfying (3) for any given INR.

The simulated KL divergence is shown in Figure 5 with randomly generated $\left\{d_{k}\right\}$ and with/without Rayleigh fading. Specifically, $h_{k}∼\exp\left(1\right)$ is an exponentially distributed variable satisfying $E[h_{k}]=1$ in Figure 5a, while $h_{k}=1$ in Figure 5b. It can be seen that, with or without Rayleigh fading, both bi-Gaussian and four-Gaussian approximation are much better than Gaussian approximation when the interference is the dominant part of the global noise (i.e., large INR). Although four-Gaussian is better than bi-Gaussian, the difference between BG and FG is much smaller than that between Gaussian distribution and true PDF. These results suggest that bi-Gaussian approximation might be sufficient.

### 3.3. LLR Calculation

Based on bi-Gaussian approximation, the LLR of each code bit can be calculated with following steps:

(1) For each codeword $x_{1},x_{2},\cdots ,x_{N}$, estimate the second and fourth moments of *y* through sample averaging of the received signal $y_{1},y_{2},\cdots ,y_{N}$, and solve the parameters μ and $\sigma^{2}$ of bi-Gaussian approximation through (12)(13).
(2) With estimated parameters $\mu ,\sigma^{2}$, calculate the LLR of the *i*-th code bit by substituting (9) into (6), namely
  (17) $$\begin{array}{cc} \lambda_{i} & =\ln\frac{p_{\mathrm{BG}}\left(y_{i}-1\right)}{p_{\mathrm{BG}}\left(y_{i}+1\right)}\\ \; & =\frac{2(y_{i}-\mu )}{\sigma^{2}}+\ln\frac{1+\exp(\frac{2\mu (1-y_{i})}{\sigma^{2}})}{1+\exp(\frac{-2\mu (1+y_{i})}{\sigma^{2}})}. \end{array}$$

Note that, if necessary, (17) can be further simplified using the methods in [50,51].

## 4. Simulation Results

In this section, we use simulation to verify the proposed BGA and compare its performance with the conventional GA.

In the simulation, we used the rate 1/3 5G LDPC code defined in [9]. The frame length of information bits was set to L=1056, and the code length was N=3168 bits. The codeword was transmitted with BPSK modulation. At the receiver, the decoder adopts the sum-product algorithm. The iterating process stops once the codeword is correctly decoded (identified by passing the parity check) and the maximum iteration number is 30. The simulation results were compared with the popular existing method (i.e., the Gaussian approximation, noting that the receiver has no information about the interfering channel or signal format) and also with the imaginary genie-aided receiver that knows the true distribution of the global noise. In the simulation, the path gain $\left\{g_{k}\right\}$ has taken into account both path loss and Rayleigh fading as in Figure 5. For both BGA and GA, the distribution parameters were estimated on a per codeword basis. For BGA, the parameters $\mu ,\sigma^{2}$ were estimated through (12)–(14). For GA, the equivalent noise power $\sigma_{z}^{2}$ was estimated by $\sigma_{z}^{2}=E\left[y^{2}\right]-1$.

### 4.1. WER Performance

**Figure 6:**
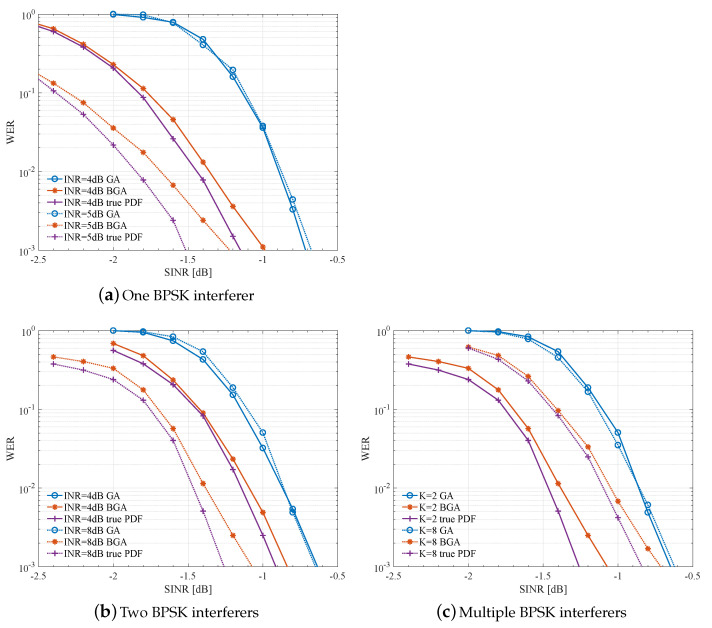
WER performance comparison using LLR calculated with BGA, GA, and exact LLR calculation under BPSK interferers.

Figure 6 compares the WER performance in the case that the interferers are BPSK modulated. The abscissa SINR and INR in Figure 6 are defined in (2) and (3), respectively.

Figure 6a shows the WER versus SINR for INR = 4.5 dB, with a single BPSK interferer in the system, i.e., K=1. In this situation, the bi-Gaussian distribution is the true distribution of global noise, and the performance loss of BGA with respect to true PDF is due to parameter estimation errors as shown in Figure 3. We can observe that, for a given SINR, the WER performance of GA is almost independent of INR, while the WER with BGA drops rapidly as INR increases. When IND=5 dB, BGA has about a 0.6 dB gain over GA. This is because, with large INR, GA will seriously deviate from the true distribution, leading to a poorly evaluated LLR that means it cannot fully exploit the non-Gaussian features. When INR = 5 dB, the loss of GA is 0.8 dB from the WER of the true PDF and the proposed BGA reduces the loss to about only 0.3 dB.

Figure 6b shows the WER versus SINR for INR = 4,8 dB, with two BPSK interferers (K=2). When INR = 8 dB, BGA has a gain of about 0.5 dB over GA, reducing the loss from 0.7 dB to less than 0.2 dB. Compared with the single interference scenario, the gain is reduced because in this situation the bi-Gaussian distribution is only an approximation of the global noise. While BGA still outperforms GA, and is close to the performance when the exact distribution is known at the receiver. The SINR gain also increases obviously when INR is large.

Figure 6c shows the WER performance with multiple BPSK interferers in the system and with fixed INR = 8 dB. Simulation shows that as *K* is increasing, BGA still has certain advantages. When K=8, BGA converges to about 0.1∼0.2 dB gain. This proves that BGA is still applicable when the components of interference are complex. In addition, the higher the proportion of the main interference, the greater the improvement of BGA, since it is easier for BGA to detect the greatest interference in the system.

**Figure 7:**
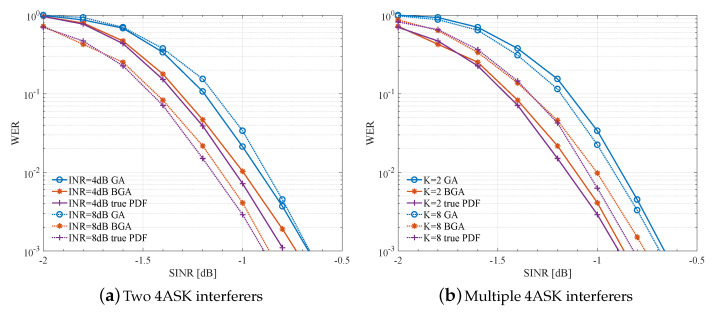
WER performance comparison using LLR calculated with BGA, GA, and exact LLR calculation under 4ASK interferers.

Figure 7 compares the WER performance of BGA, GA, and the true PDF in the case that the interferers are 4ASK modulated.

Figure 7a presents the WER performance with two 4ASK interferers. BGA shows 0.1∼0.3 dB gain under several given INRs, and with INR increasing, the gain still has a positive growth trend. The loss of BGA is only 0.02∼0.08 dB from the WER of the true PDF. Compared with the case of BPSK interferers, the gain decreases because, for high-order modulations, bi-Gaussian distribution is also a rough approximation of the global noise, but BGA still outperforms GA since it takes into account the high-power components in the global noise, while just the estimated $\mu^{2}/\sigma^{2}$ will be smaller than the actual INR. Therefore, BGA also suits high-order modulations, and the gain is more obvious in high-INR scenarios.

Figure 7b presents the WER performance with multiple 4ASK interferers in the system and with fixed INR = 8 dB, representing the complex scenario of multiple interferers with high-order modulations. In this case, the gain is about 0.1 dB, which means that BGA has only a little improvement in WER performance. Although the gain of BGA on WER performance reduces under complex interference, the advantage still exists and is highly obvious in decoding complexity as shown in the next section.

### 4.2. Complexity Analysis

**Figure 8:**
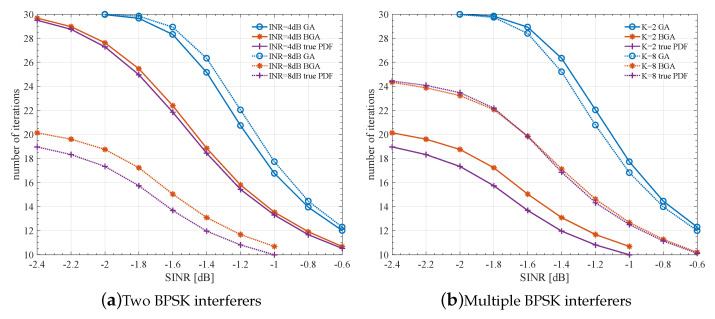
Decoding complexity comparison using LLR calculated with BGA, GA, and exact LLR calculation under BPSK interferers.

The improved LLR can accelerate the convergence of iterative decoding and hence reduce the overall decoding complexity. Figure 8 compares the average iteration number versus SINR under different global noise models (BGA, GA, true PDF), different INRs, and different numbers of interferers. We can see that the proposed BGA can significantly reduce the complexity, and the number of iterations can be reduced by 25∼50% for the SINR range of interest. Note that with the proposed BGA, calculating LLR through (17) will introduce some extra complexity and this part is not included in Figure 8. The calculation involved in (17) per code bit is roughly similar to the updating of a check node of degree 3. For the LDPC code used in this paper, the aggregate weight of the parity matrix is 4.65 per code bit. Hence the extra complexity introduced by (17) is negligible to the overall decoding complexity.

## 5. Conclusions

This paper focuses on LLR calculation under non-Gaussian global noise with an unknown distribution. This situation is common in multi-user interfering scenarios. Since the true distribution of global noise is unknown, most of existing systems treat the interference plus noise as Gaussian. GA has the advantage of very low complexity, but the approximation may be inaccurate and hence lead to performance loss. In this paper, we proposed an improved LLR estimation using bi-Gaussian distribution to approximate the global noise. The parameters of the BGA can easily be estimated from the second and fourth moments of the received signals, without the knowledge of CSI and MCS information of interfering signals. Compared with conventional GA, BGA is closer to the real global noise, especially when INR is large. With the LLR estimated with BGA, the decoder can improve the WER performance and accelerate the convergence of iterative decoding.

## Data Availability

The data presented in this study are available on request from the corresponding author.
